# Simulated Anthrax Attacks and Syndromic Surveillance

**DOI:** 10.3201/eid1109.050223

**Published:** 2005-09

**Authors:** James D. Nordin, Michael J. Goodman, Martin Kulldorff, Debra P. Ritzwoller, Allyson M. Abrams, Ken Kleinman, Mary Jeanne Levitt, James Donahue, Richard Platt

**Affiliations:** * HealthPartners Research Foundation, Minneapolis, Minnesota, USA;; † Harvard Medical School, Boston, Massachusetts, USA;; ‡ Harvard Pilgrim Health Care, Boston, Massachusetts, USA;; § Kaiser Permanente, Boulder, Colorado, USA;; ¶ Marshfield Clinic Research Foundation, Marshfield, Wisconsin, USA

**Keywords:** Anthrax, bioterrorism, managed care programs, Minnesota, statistical models, population surveillance, research

## Abstract

We measured sensitivity and timeliness of a syndromic surveillance system to detect bioterrorism events. A hypothetical anthrax release was modeled by using zip code population data, mall customer surveys, and membership information from HealthPartners Medical Group, which covers 9% of a metropolitan area population in Minnesota. For each infection level, 1,000 releases were simulated. Timing of increases in use of medical care was based on data from the Sverdlovsk, Russia, anthrax release. Cases from the simulated outbreak were added to actual respiratory visits recorded for those dates in HealthPartners Medical Group data. Analysis was done by using the space-time scan statistic. We evaluated the proportion of attacks detected at different attack rates and timeliness to detection. Timeliness and completeness of detection of events varied by rate of infection. First detection of events ranged from days 3 to 6. Similar modeling may be possible with other surveillance systems and should be a part of their evaluation.

## Abstract 2

Bioterrorism surveillance systems can be assessed using modeling to simulate real-world attacks.

Numerous syndromic surveillance systems are in place to detect potential bioterrorism events, and all of them have common components: a nonspecific indicator of disease available in near real time for a definable population, a means of generating the expected counts for each day of the year (accounting for day of week and seasonal variability), a detection algorithm, a defined threshold for action, and a system to investigate a signal. Assessing the sensitivity and timeliness of these systems has been difficult. Because of the lack of real events, modeling of hypothetical events is necessary to assess the performance of these systems. Although existing systems can be assessed by using naturally occurring illness events such as the beginning of the influenza season each year or gastrointestinal outbreaks, this assessment provides little information as to how well these systems will detect the release of a bioterrorism agent such as anthrax. Buehler et al. (*1*), Sosin and de Thomasis (*2*), and Reingold (*3*) have called for additional assessment of syndromic surveillance systems.

The existing literature on the ability of syndromic surveillance systems is sparse. Mandl et al. used a purely temporal approach with real background data and simulated spiked outbreaks (*4*). They described a variety of ways to design the spiked data, including specifically using data from the Russian anthrax release in 1979 (*5*). They describe 4 steps in detection: grouping data into syndromes; the modeling stage, in which historic data are studied to understand temporal trends; the detection stage, in which predictions based on the modeling are compared with observed data and deviations of data from expectations are used to set off alarms; and the threshold stage, in which the health department determines if the outbreak is worth investigation. Buckeridge et al. describe a complex model to produce a realistic space-time simulation of spiked outbreaks of anthrax superimposed on real background data (*6*). Their model of a simulated event has 4 stages: agent dispersal, infection, disease and behavior, and data source. Kulldorff et al. produced a testing data set with simulated space-time outbreaks and simulated background data (*7*). Within these data sets, positive predictive value, sensitivity, and specificity can be evaluated for a variety of testing systems that analyze both purely temporal and temporal-spatial aspects of outbreaks.

None of these efforts has used a functioning system to analyze how effective it would be at detecting a biological attack. This article presents the first attempt to evaluate the performance of an operational syndromic surveillance system to detect a bioterrorism attack in a quantitative and rigorous manner and provides a useful construct for other systems to follow. A comparison of our methods with those used in previous bioterrorism surveillance assessments is shown in Table 1.

**Table 1:** Methods used in bioterrorism surveillance assessments

Study (Reference)	Epidemic data	Background data	Analysis	Surveillance system
Mandl et al. (*4*)	Simulated	Real	Temporal	Nonfunctional
Buckridge et al. (*6*)	Detailed simulation	None	Spatial-temporal	None
Kulldorff et al. (*7*)	Simulated	Simulated	Spatial-temporal	Nonfunctional
Nordin et al. (this study)	Simulated	Real	Spatial-temporal	Functioning system

## Methods

This syndromic surveillance system is part of the National Syndromic Surveillance System (*8**,**9*). This current investigation is based on existing historic use data contributed by the HealthPartners Medical Group (HPMG). We assumed that anthrax spores were released into one of the air intakes of the Mall of America in Bloomington, Minnesota, and then dispersed by the ventilation system, which provided a uniform exposure throughout the mall. Modeled visits of patients with respiratory symptoms were produced by using 3 factors: demographic data from the mall, demographic data on HPMG patients, and data on time to symptom onset from the anthrax outbreak in Sverdlovsk (*5*). Historic data were used to add the number of respiratory cases that would be expected under ordinary conditions. Finally, the detection sensitivity and timeliness of the system were analyzed.

### Syndromic Surveillance Data

HPMG provides medical care in 20 clinics to ≈250,000 patients, or ≈9% of the total population in the Minneapolis-St. Paul, Minnesota metropolitan area. It uses an electronic medical record that captures in real time nearly all physician visits and makes them accessible at the end of each day. Each evening, the data system is queried to obtain all visits for respiratory symptoms for that day. Respiratory symptoms were used to build the model for this simulation. One year of this dataset, from July 1, 2003, through June 30, 2004, was used to test this project. We had 2.5 years of earlier data, which allowed us to establish the number of visits that would ordinarily be expected.

### Anthrax Simulation Model

The number and geographic distribution of patients with anthrax were modeled by using visitor data from the mall, the US Census bureau (http://www.census.gov/geo/www/gazetteer/places2k.html), and demographic data from HPMG. We purposely chose to model the first 3 stages of infection of Buckeridge et al. (*6*) (agent dispersion, infection, and disease and behavior) as a simple rate of visits for respiratory illness because those data were available from the Sverdlovsk outbreak, and they produced a simpler model. Keeping the model simple is initially important to allow the basic relationships between the variables to be understood. The rate of physician visits for respiratory symptoms ranged from 4% to 100% of the visitors to the mall that day. The specific rates for which models were run were as follows: 4%, 8%, 12%, 16%, 20%, 40%, 60%, and 100%.

The Sverdlovsk outbreak generated no physician visits on day 1; the number of visits increased until day 9 and then decreased. We created a cumulative distribution of the probability of a visit for respiratory symptoms each day from day 0 to day 30. We did not run the simulation beyond day 30 because detecting an outbreak with our system would not be beneficial at that point. To prevent a continuous signal pattern, we introduced variation by using a Poisson distribution consistent with the cumulative distribution. This distribution simulates the natural variation that would be expected in such scenarios.

We used the following approach. First, we created a cumulative distribution of the respiratory visits expected each day from 1 through 30. For example, the cumulative distribution was 0.0 for day 1, 0.01 (0.00 + 0.01) for day 2, 0.03 (0.00 + 0.01 + 0.02) for day 3, etc. Second, we assigned a random number from 0.0 to 1.0 from a uniform distribution as each randomly created day for the number of infections. Third, if the random number generated was between the minimum cumulative range for a day and the maximum cumulative range for a day, we then produced a new visit from the infection. All calculations were rounded down to the nearest integer. The effect is shown in Table 2, which shows how many visits occurred during the simulations from 1 zip code on day 6. Of the 1,000 simulations, no visits occur 123 times on day 6. Four times, however, 8 visits occur. Most of the time 1, 2, or 3 visits occur on day 6.

**Table 2:** Visit distribution of 1,000 simulations at a 40% infection rate for day 6 from infection for zip code 55125 (St. Paul, Minnesota)

No. visits on day 6	Frequency
0	123
1	258
2	274
3	191
4	97
5	41
6	5
7	7
8	4

Each of the 1,000 simulations at the given infection rate was randomly assigned (with replacement) to an attack date. The additional cases were added to the historic data based on the date randomly chosen for each iteration, which created 1,000 new files.

The expected number of visits added for a specific release can be expressed as
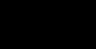
where *n* = the number of zip codes in the outbreak, *m_i_* = number of mall visitors in zip code *i*, *h_i_* = number of HealthPartners patients in zip code *i*, *d* = infection rate of the simulation, and *pop_i_* = population of zip code *i*. The actual number of added cases was random and followed Poisson distribution centered on the expected count.

This equation allows us to vary the infection rate of the attack and the number of patients from the zip code. With a limited number of simulations, this variation allows us to assess the effect of an attack with a variety of infection rates and rates of HPMG's penetration in the community and the zip code.

### Release Detection Method

The Poisson-based prospective space-time scan statistic was used to detect releases (*10*). This method uses a large number of overlapping cylinders in which the height of the cylinder represents time and the circular base represents space in such a way that all zip code areas whose centroid (the population-weighted geographic center of the area) is within the circle are included in the cylinder. Each cylinder represents a candidate area and duration for a true disease outbreak, and the method adjusts for the multiple testing inherent in the many cylinders evaluated. We evaluated all cylinders for which the circle center was identical to the centroid of one of the zip code areas; for each circle center the maximum radius of the circle was set so that <50% of the at-risk population was contained in the zip codes included within the circle, and the height of the cylinder was set to be <3 days. Purely temporal cylinders, including all zip code areas and either 1, 2, or 3 days, were also evaluated. The method needs expected counts for each day and zip code, and these were determined on the basis of historical data from January 1, 2001, to June 30, 2003, by using a generalized linear mixed modeling approach that accounts for natural seasonal and weekly variation in the data (*11*). Analyses were performed by using the freely available SaTScan software (http://www.satscan.org).

A signal is detected when the number of episodes of respiratory illness is substantially greater than expected. The rarity of an outbreak signal is measured as a recurrence interval, which is defined as the expected number of days of surveillance needed for a signal of at least the observed magnitude to occur, in the absence of any true outbreaks, and it is the inverse of the nominal p value from the space-time scan statistic. Thus, the larger the recurrence interval, the more unusual the outbreak signal. We present results that use recurrence intervals of 3 months (p = 0.011) and 2 years (p = 0.0013). At the 3-month recurrence interval level, an ≈4 positive signals will occur per year by chance, whereas at the 2-year level, only 1 positive signal expected by chance will occur every 2 years.

For each simulated attack, the spiked data were analyzed for each of the 10 days after the attack. We then report the proportion of all attacks that generated an outbreak signal on or before each of days 2 to 10.

## Results

Timeliness and completeness of detection of events varied by rate of infection and by percentage of population covered by HPMG. Initial models were done at the current 9% population coverage. At a 40% infection rate and a recurrence interval of 3 months (p = 0.011), the first events (outbreaks) were detected on day 2, one fourth by day 6, three fourths by day 7, and all 1,000 by day 8. With higher percentages of infections, all events were detected earlier. At an infection rate <40%, not all events were detected. At a 20% infection rate, 845 of 1,000 events were detected at a 2-year recurrence interval, and 926 of 1,000 events were detected at a 3-month recurrence interval (both peaking at day 8). The number of events detected decreases proportionately as the infection rate decreases from 20% to 4%. At a 4% infection rate little is detected; 7 events are detected at a 2-year recurrence interval, and 57 events are detected at a 3-month recurrence interval.

At a 16% infection rate, more events were detected during the summer than during the winter, with an intermediate number of events detected in the fall and spring (Table 3). The day of release also affected the number of events detected. When the number of cases peaked on Saturday or Sunday, more events were detected (Table 4). The number of days until detection peaked increased as the rate of infection decreased.

**Table 3:** Number of releases detected by season at a 16% infection rate

Season of release	No. releases	No. detected (%)
Winter	248	131 (52.8)
Spring	276	204 (73.9)
Summer	189	165 (87.3)
Fall	287	196 (68.3)

**Table 4:** Number of releases detected by day of week at a 16% infection rate*

Day of release	No. releases	No. detected (%)
Sunday	146	146 (100.0)
Monday	132	64 (48.5)
Tuesday	142	50 (35.2)
Wednesday	142	63 (44.3)
Thursday	156	100 (64.1)
Friday	143	134 (93.7)
Saturday	139	139 (100.0)

*Most outbreaks at this level are detected 7 or 8 days later. Thus, for outbreaks starting on Saturday or Sunday, more are detected during the next weekend.

At infection rates >16%, the relationship of detection to season was weaker because all events were detected. However, events are detected more rapidly in the summer when respiratory conditions are less common. The relationship of the day of the week with release is more complex. As the infection rates increase to >40%, the number of days until all events are detected gets smaller, and the highest rate of detection occurs closest to the time of release. Events were best detected when the highest rates of visits occurred on the weekend.

The percentage of the population in the area covered by the surveillance system directly affects the smallest size of outbreaks that can be reliably detected and the timeliness of detection of those outbreaks. We also computed system characteristics if 36% of the population (4 times as much) were covered by the surveillance system. In this situation, at a recurrence interval of 3 months (p = 0.011) and an infection rate of 50%, all events were detected by day 3 and most by day 2. At a 10% infection rate, the first events were detected on day 2, one fourth by day 6, three fourths by day 7, and all 1,000 events by day 8.

**Figure 1:**
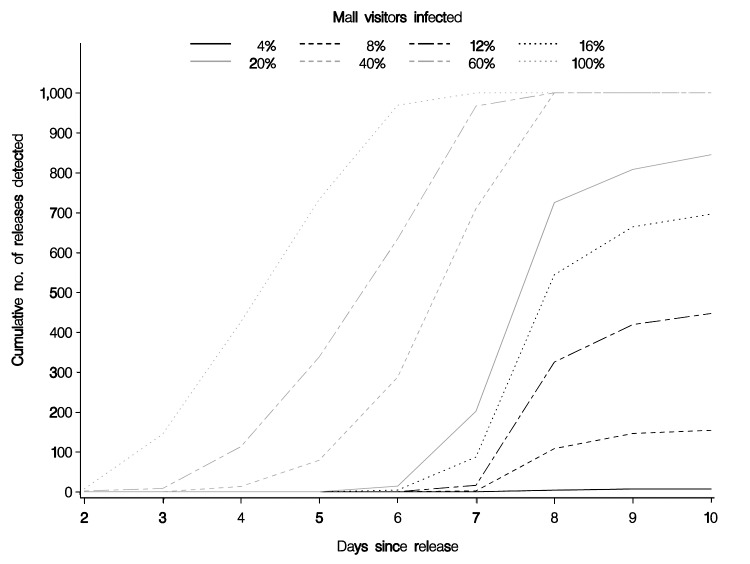
Cumulative number of releases detected in a recurrence interval of 2 years (p = 0.0013) with 9% of the population covered.

**Figure 2:**
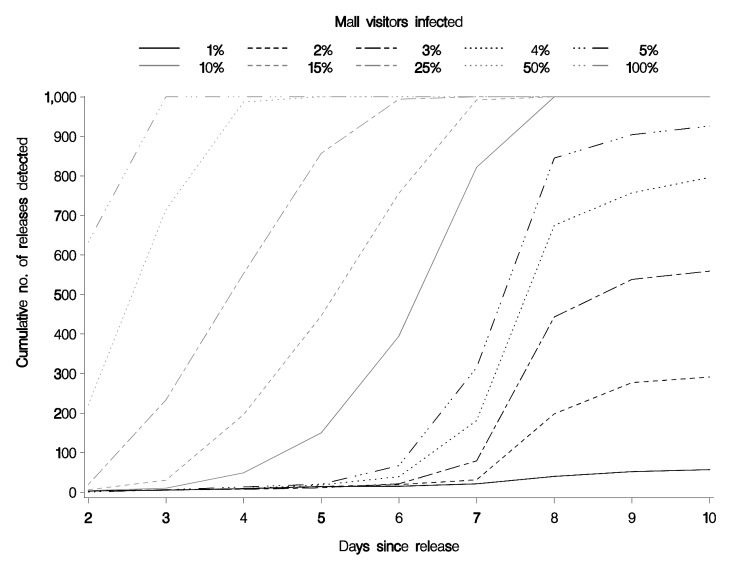
Cumulative number of releases detected in a recurrence interval of 3 months (p = 0.011) with 36% of the population covered.

The extremes of sensitivity and timeliness are shown in Figures 1 and 2. The first extreme, with a high threshold and 9% of the population, shows the lowest sensitivity, while the second extreme, with a low threshold and 36% of the population, shows high sensitivity. Most configurations would fall somewhere between these 2 extremes.

## Discussion

This study has several limitations. Simplifying assumptions was crucial to the construction of this simulation and are both its strength and weakness. We assumed that most patients would have respiratory symptoms. The first 3 stages of the model of Buckeridge et al. (*6*) are merged. The distribution of intervals from exposure to initial observance of disease we used was based on incubation periods determined from the Sverdlovsk outbreak in Russia (*2*).

The inadvertent release of anthrax spores from a military microbiology facility in Sverdlovsk, Russia, in 1979 is the largest documented epidemic of inhalational anthrax and clearly demonstrates the potential for *Bacillus anthracis* to be used as a weapon. In Sverdlovsk, the spores were likely released on a single day. The incubation period of anthrax in this outbreak ranged from 2 to 3 days to slightly more than 6 weeks; the modal incubation period was 9–10 days. Other investigators have used the Sverdlovsk data to compute a median incubation period of 11 days (*12*).

Some patients will likely have initial symptoms only a few days after the exposure, and their conditions diagnosed a few days later. Thus, a suspicious clinician may detect the first case of anthrax before the surveillance system sounds an alarm and public health determines it is an anthrax release. However, even if the first alarm is sounded by a clinician, these additional data will help define what is happening and plan a response. This simulation exercise could be applied to a surveillance system in any metropolitan area where gathering place is different enough from residence so that exposed persons would live far from each other.

The sensitivity of such a system in detecting small releases of anthrax depends on the proportion of the population covered by the system. The greater the proportion of the population covered by such a system, the more sensitive it is. According to this model, a system that includes 36% of the population in the area would detect most events in which >5% of mall shoppers were affected.

Outdoor releases, similar to the outbreak in Sverdlovsk, have been modeled previously. These models produce marked geographic clustering, with some spread from persons who pass through the area. In a large regional shopping center that draws people from large areas, detection is more difficult. Because infected persons live far from each other, a larger number of cases were needed to detect the outbreak in our model than in earlier models of outdoor releases.

The relationship between the days of the week and detection of events is complex. Fewer patients visit the clinic on weekends since only 4 urgent-care clinics are open instead of the usual 20. As the rate of infection increases, the maximum number of events detected occurs more quickly. When the maximum number of events detected occurs on weekends, the system is more sensitive. Thus, as the rate of infection increases, the day of the week for the release with the most sensitivity shifts closer to the weekend.

Simulation modeling is necessary to test and prepare syndromic surveillance systems. Although more complex simulation modeling can be done, it requires more assumptions and may be more sensitive to error because of the additional risk of false assumptions.

## Conclusions

This article reports the evaluation of an operational bioterrorism surveillance system. This analysis allows an understanding of limitations of the system and characteristics unique to the region the surveillance system is monitoring. The HPMG bioterrorism surveillance system, which receives data for ≈9% of the population in the area, can detect an anthrax release in the Mall of America most of the time if 20% of the persons at the mall at the time of release are infected and all of the time at a 40% infection rate. The time to detection gets progressively shorter as the infection rate increases >40%. Modeling with 36% population coverage showed that such a system would be capable of detecting a release at a 5% infection rate most of the time and at a 10% infection rate all the time. Similar modeling may be possible with other surveillance systems and should be used as a part of their evaluation.
